# Quantifying the effects of drought on abrupt growth decreases of major tree species in Switzerland

**DOI:** 10.1002/ece3.2146

**Published:** 2016-04-20

**Authors:** Marco Vanoni, Harald Bugmann, Magdalena Nötzli, Christof Bigler

**Affiliations:** ^1^ Forest Ecology Institute of Terrestrial Ecosystems Department of Environmental Systems Science ETH Zurich Universitätstrasse 16 CH‐8092 Zurich Switzerland

**Keywords:** *Abies alba*, breakpoint detection, distributed lag nonlinear models, *Fagus sylvatica*, *Picea abies*, *Quercus* spp., structural changes, tree rings

## Abstract

Drought entails important effects on tree physiology, which may result in short‐ to long‐term radial growth decreases. While the majority of studies have focused on annual drought‐related variability of growth, relatively little is known about sustained growth decreases following drought years. We apply a statistical framework to identify climatic factors that induce abrupt growth decreases and may eventually result in tree mortality. We used tree‐ring data from almost 500 standing dead trees and 200 living trees in eight sites of the Swiss network of strict forest reserves, including four of the most important Central European tree species (*Abies alba*,* Picea abies*,* Fagus sylvatica* and *Quercus* spp.). First, to assess short‐term growth responses to drought under various climate and site conditions, we calculated correlations and linear mixed‐effects models between ring‐width indices (RWIs) and drought based on the Standardized Precipitation Evapotranspiration Index (SPEI). Second, to quantify drought effects on abrupt growth decreases, we applied distributed lag nonlinear models (DLNMs), which account for both delayed effects and the nonlinear relationship between the SPEI and the occurrence of abrupt growth decreases. Positive correlations between RWIs and the SPEI indicated short‐term growth responses of all species, particularly at arid sites. Results of the DLNMs revealed species‐specific growth responses to drought. For *Quercus* spp., abrupt growth decreases were more likely to occur several years following severe drought, whereas for *P. abies*,* A. alba,* and *F. sylvatica* abrupt growth decreases started frequently immediately in the drought year. We conclude that the statistical framework allows for quantifying the effects of drought intensity on the probability of abrupt growth decreases, which ultimately contributes to an improved understanding of climate impacts on forest community dynamics.

## Introduction

Drought‐induced tree mortality has become an important topic in many regions worldwide (Allen et al. 2010; Anderegg et al. 2013; Rigling et al. 2013). In the Fifth Assessment Report of the Intergovernmental Panel on Climate Change, many drought‐induced forest declines and tree mortality events were reported, covering forest regions around the globe (Settele et al. 2014). Under global climate change, the frequency and severity of droughts are expected to increase not only in arid areas but more and more in mesic areas, where prolonged droughts have not played a major role to date (Beniston et al. 2007; Zang et al. 2014; Martin‐Benito and Pederson 2015). Since forests are important terrestrial ecosystems in terms of carbon stocks, drought‐induced forest declines could have major consequences for the global carbon cycle (Anderegg et al. 2015). Impacts of extreme climatic events on ecosystems such as drought effects on forest health are not necessarily instantaneously apparent but are often lagged by several years (Reichstein et al. 2013). Therefore, an improved understanding of drought effects on forest community dynamics is essential.

Tree mortality is a key process in forest dynamics. Complex interactions between multiple ecological processes lead to high temporal and spatial variability of tree mortality (Franklin et al. 1987; Wunder et al. 2007). Several types of mortality triggers result in “background mortality” (Hawkes 2000; Keane et al. 2001), such as neighborhood interactions that determine the competitiveness of adjacent trees (Weber et al. 2008; Krumm et al. 2012), or age‐related mortality due to declining vigor and resistance (Lines et al. 2010). In contrast to these local and often tree‐specific mortality events, disturbance‐induced mortality caused, for example, by windthrow or drought drives mortality processes at the stand or regional scale (Keane et al. 2001). Extreme climatic events such as severe drought may initiate synchronous short‐ to long‐term growth decreases, which may ultimately lead to tree death. However, the occurrence of lagged growth or mortality responses to climate anomalies still represents a challenge in detecting the actual trigger of the response (Bigler et al. 2007; Babst et al. 2012). Mortality rates have been observed to increase immediately after drought periods (e.g., Solberg 2004 or Linares and Camarero 2012) or after a lag of several years (e.g., Bigler et al. 2007 or Suarez and Kitzberger 2010). Under global climate change, trees are expected to increasingly suffer either from a lack of precipitation or from increased evaporative demand (Williams et al. 2013). Thus, quantifying the effects of drought periods on the radial growth patterns of trees is of high importance to understand and project forest community dynamics.

Growth−climate relationships have been widely studied by correlating tree‐ring indices from a chronology with climate variables such as precipitation, temperature, or drought (Fritts 1976). Typically, a set of tree‐ring series from one species within a site or region is aggregated and standardized to a tree‐ring chronology that comprises a population signal. For example, significant mean growth responses to drought have been identified for several Central European tree species (Weemstra et al. 2013). However, the impact of climatic factors at the level of individual trees has been analyzed less often. As suggested by Carrer (2011), the prevalent focus on the mean growth response to climate variability across a sample of trees at the local to regional scale should shift toward the investigation of tree‐specific responses. Several recent studies have considered growth−climate relationships at the level of individual trees (e.g., Rozas and Olano 2013 for *Juniperus thurifera*, Galván et al. 2014 for *Pinus uncinata* or Rozas 2015 for *Quercus robur*).

There is ample evidence that slow growth in combination with negative growth trends indicates an increased mortality risk (Bigler and Bugmann 2003; Bigler et al. 2006). Thus, it is important to evaluate the link between the onset of a period with reduced growth and a potential inciting factor (e.g., severe drought). Unlike correlations between mean growth responses and climate variables, relatively few studies have focused on the effects of extreme climatic events on tree growth (e.g., Pedersen 1998; Lloret et al. 2011; Zang et al. 2014). Recently, some tree‐ring‐based studies have attempted to identify severe drought as an inciting factor of tree decline and dieback, for example, based on the approach of early warning signals (Camarero et al. 2015; Mamet et al. 2015) or on the comparison of individual ring‐width indices (RWIs) with a site chronology (Amoroso et al. 2015).

The objective of the present study is to quantify the response of radial growth to drought periods of several months for four major tree species of Central Europe in unmanaged forests in Switzerland. The growth responses are tested at sites distributed along a drought gradient. We focus on the one hand on the short‐term growth response at the individual‐tree and population level, and on the other hand on the longer term growth response at the individual‐tree level. In particular, we expand the currently used methods to relate extreme climate events to tree decline with a statistical framework that allows us to identify abrupt growth decreases and relate the occurrence of these growth decreases to drought intensity, accounting for both lagged and nonlinear effects. We hypothesize that (1) drought is a major driver of short‐term growth response of trees; (2) abrupt longer term growth decreases frequently emerge during and shortly after severe drought; and (3) there are species‐specific short‐ to long‐term growth responses to drought across sites.

## Material and Methods

### Study area and species

We selected eight reserves of the Swiss network of strict forest reserves, that is, Weidwald, Scatlè, Bois de Chênes, Vorm Stein, Tariche Haute Côte, Leihubel, Strassberg, and Combe Biosse (Table 1) that had been established by ETH Zurich between 1965 and 1987 and were not managed any more after the establishment (Brang et al. 2011). Some forests had not been managed for several decades up to centuries (e.g., primeval spruce forest “Scatlè”) prior to reserve establishment. In each reserve, a variable number of permanent plots with a size of up to 1 ha had been installed, and in the permanent plots each tree above a minimum diameter at breast height (DBH) of 4 cm was tagged. Several variables such as species, DBH, or tree status (dead/alive) were recorded repeatedly at intervals of 5–10 years.

**Table 1:** Characteristics of selected forest reserves. Species: beech (*Fagus sylvatica*), spruce (*Picea abies*), oak (*Quercus* spp.), fir (*Abies alba*)

Forest reserve code	Site	Coordinates WGS84	Species	Mean elevation (m a.s.l.)	Average temperature (°C)	Precipitation sum (mm)
11	Weidwald	47°24′53″N 7°59′47″E	Beech	550	8.32	1163
12	Scatlè	46°47′26″N 9°2′54″E	Spruce	1600	3.29	1582
14	Bois de Chênes	46°26′8″N 6°14′20″E	Beech, oak, spruce	530	9.59	1042
20	Vorm Stein	47°33′10″N 8°27′13″E	Oak	540	8.65	1118
22	Tariche Haute Côte	47°20′08″N 7°9′43″E	Beech, fir	750	7.76	1228
24	Leihubel	46°52′8″N 8°8′35″E	Spruce, fir	1200	5.96	1711
30	Strassberg	47°31′55″N 8°29′45″E	Oak	470	8.83	1068
39	Combe Biosse	47°6′21″N 7°0′38″E	Fir	1250	5.88	1363

Climate regimes in Switzerland are heterogeneous, which are characterized by oceanic, insubric, or continental conditions and steep topographical gradients leading to variations of the climate conditions among the forest reserves. The reserves Weidwald, Bois de Chênes, Vorm Stein, and Strassberg that are located in the Swiss Plateau show fairly similar conditions with warm summer and annual precipitation of around 1100 mm; in Tariche Haute Côte and Combe Biosse in the subalpine Jura Mountains the amount of annual precipitation is ~200 mm higher; in Scatlè in the inner‐alpine region of the Alps annual precipitation is >1600 mm; and in Leihubel located in the subalpine Swiss Prealps >1700 mm of annual precipitation is recorded. Temperatures are mainly dependent on the altitude, with values ranging between 3.29°C at the highest site Scatlè and 9.59°C in Bois de Chênes in western Switzerland.

We selected four species that are major Central European temperate tree species: Norway spruce (*Picea abies* [L.] Karst.), silver fir (*Abies alba* Mill.), European beech (*Fagus sylvatica* L.) and oak (*Quercus* spp.), the latter including both sessile oak (*Quercus petraea* Liebl.) and pedunculate oak (*Q. robur* L.), hereafter referred to as spruce, fir, beech, and oak. Although distinction between the two oak species based on leaf or acorn morphology is possible, the wood anatomical characteristics at the macroscopic and microscopic level do not allow clear differentiation (Hroš and Vavrčík 2014). In Bois de Chênes, most of the living oaks were identified as *Q. robur*, while a majority of the living oaks in Vorm Stein and Strassberg showed typical leaf and acorn characteristics of *Q. petraea*. Because of the absence of leaves on the dead trees and since both species had been recorded as *Q. robur* in the inventories, *Q. robur* and *Q. petraea* are denoted here as “oak” (*Quercus* spp.).

For mature trees, oak is considered to be more anisohydric (drought‐tolerant) than the other three investigated species, which show a decreasing drought tolerance from fir to beech and the most isohydric spruce (Ellenberg and Leuschner 2010). Spruce, as the economically most important tree species in Europe, is attested a high vulnerability to drought compared to beech and fir (Pretzsch et al. 2013; Zang et al. 2014). Prior work has documented a high vulnerability of fir and beech to late frost events, which do not affect the other species to the same degree (Ellenberg and Leuschner 2010). Both fir and beech have a high shade tolerance, followed by spruce and oak (Ellenberg and Leuschner 2010).

### Field sampling

The field campaign was conducted in summer and autumn 2013 before leaf fall. In one reserve, three of the four target species were abundant; in two reserves, two target species were present; and in five reserves, only one target species could be sampled (Table 1). The mean elevation of the sampling sites in the reserves ranged from 470 to 1600 m a.s.l. From each of the four species, 150 standing dead trees (i.e., snags) were sampled, 50 trees each from three different sites, resulting in a total of 12 sampling groups. Tree selection was based on the DBH distribution in the last inventory to obtain a representative sample of the forest stand, that is, trees over the entire DBH range were selected in proportion to their abundance (cf. Fig. S1). A tree was classified as “dead” if green leaves and needles, respectively, were lacking. In a first step, snags recorded in the last inventory were searched. If no apparent damages or wounds (e.g., rockfall scars, broken trunk etc.) were observed and if the trunk appeared intact at a height of 1 m, the tree was selected for sampling. If no further tagged snags from the last inventory could be found, untagged snags within or outside the permanent plots were selected based on the same criteria (lack of green leaves/needles, no apparent damage, intact trunk). From each dead tree, two increment cores were extracted at a height of 1 m parallel to the contour lines to reduce effects of eccentricity caused by compression and tension wood, respectively. Further measurements were recorded including DBH and tree social status. In total, 1200 increment cores from 600 dead trees were collected.

Site chronologies were available for the oak sites (Rohner et al. 2013) and one spruce site (Bigler and Bugmann 2003). In reserves without an existing tree‐ring chronology, 12 dominant living trees were sampled each by extracting one increment core at breast height (1.3 m). In total, 100 increment cores from living trees were collected, and 100 increment cores from these earlier studies could be used.

### Dendrochronological methods

Increment cores were air‐dried, glued on core mounts and then progressively sanded or cut with a core microtome (Gärtner and Nievergelt 2010). Ring widths were measured to the nearest 0.01 mm on a Lintab 5 measuring system using the TSAP‐Win software (RINNTECH, Heidelberg, Germany). The tree‐ring series were crossdated visually and statistically checked with the software COFECHA (Holmes 1983). Crossdating of the dead trees was performed based on site chronologies that were built from the sampled living trees and available site chronologies, respectively. Due to partial cambial mortality, the formation year of the outermost tree ring from the two cores of the same trees differed in several trees. In addition, we identified several wedging or missing rings, mainly toward the end of the series. Thus, dead trees were excluded from the further analyses if crossdating of both increment cores failed due to missing or wedging rings that could not be identified. As a result, 101 dead trees (16.8% of the sample) were excluded, and, if both cores were crossdated from any of the remaining 499 trees, only the better preserved core was used for further analysis (Table 2). Year‐of‐death dates were estimated based on the formation year of the outermost tree ring that was present either on one or the other increment core.

**Table 2:** Tree‐ring characteristics of the living and dead trees. Living trees represent the dominant trees of the stand; dead trees represent the complete range of social positions, that is, from suppressed to dominant trees. Number of trees per sampling group corresponds to crossdated trees. The standard deviation of life span and growth rate is denoted as “SD”

Sampling group^1^	Site	Living trees			Dead trees		
		Number	Average life span ± SD (years)	Average growth rate ± SD (mm/year)	Number	Average life span ± SD (years)	Average growth rate ± SD (mm/year)
Spruce 12	Scatlè	15	155.2 ± 58.2	0.68 ± 0.27	35	168.3 ± 70.9	0.76 ± 0.30
Spruce 14	Bois de Chênes	18	80.6 ± 10.0	1.97 ± 0.73	35	60.9 ± 9.8	1.15 ± 0.33
Spruce 24	Leihubel	11	199.4 ± 52.6	1.45 ± 0.55	29	128.9 ± 63.6	0.78 ± 0.35
Fir 22	Tariche Haute Côte	11	122.5 ± 15.4	1.93 ± 0.51	36	88.8 ± 15.0	0.84 ± 0.50
Fir 24	Leihubel	12	160.6 ± 66.3	1.75 ± 0.82	38	82.1 ± 38.8	0.76 ± 0.27
Fir 39	Combe Biosse	12	133.9 ± 31.0	1.77 ± 0.30	44	92.9 ± 28.0	0.88 ± 0.46
Oak 14	Bois de Chênes	27	103.5 ± 27.8	1.17 ± 0.44	50	119.2 ± 34.2	0.80 ± 0.21
Oak 20	Vorm Stein	30	87.6 ± 7.3	1.37 ± 0.46	50	93.0 ± 21.2	0.81 ± 0.21
Oak 30	Strassberg	29	80.1 ± 25.1	1.27 ± 0.27	47	78.4 ± 23.8	1.19 ± 0.26
Beech 11	Weidwald	11	145.6 ± 9.2	1.03 ± 0.31	47	120.6 ± 18.7	0.86 ± 0.31
Beech 14	Bois de Chênes	12	82.6 ± 20.3	2.14 ± 0.97	44	75.1 ± 15.5	1.14 ± 0.60
Beech 22	Tariche Haute Côte	12	131.7 ± 7.6	1.72 ± 0.19	44	121.6 ± 27.1	0.86 ± 0.22

^1^Species and forest reserve code (cf. Table 1).

### Climate data and drought index

We used monthly total precipitation and mean temperature from 1931 to 2011 that were spatially interpolated across Switzerland on a grid with 1 ha grid cells based on the DAYMET model (Thornton et al. 1997) at the Swiss Federal Institute for Forest, Snow and Landscape Research WSL (Birmensdorf). Climate data for the interpolation originated from climate stations of MeteoSwiss (Federal Office of Meteorology and Climatology). For each site, we averaged the precipitation and temperature data from nine surrounding grid cells. As an indicator for drought, we used the Standardized Precipitation Evapotranspiration Index (SPEI), a multiscalar drought index that incorporates both precipitation and temperature (Vicente‐Serrano et al. 2010). Negative SPEI values indicate dry conditions; positive SPEI values indicate moist conditions. To estimate potential evapotranspiration, we used the Thornthwaite method, which considers average day length and mean temperature (Thornthwaite 1948). The SPEI was calculated for 1‐, 3‐, 6‐, and 12‐month time scales using the SPEI package (Beguería et al. 2014) in the statistical software R (R Core Team 2015).

### Short‐term growth response to climate

To quantify the short‐term effects of climate on variability of tree growth, residual chronologies of the living trees were built. First, dimensionless RWIs of the living trees were calculated using a 32‐year spline detrending with a frequency response of 50%. Second, the autocorrelation was removed by autoregressive modeling. Third, the residuals were averaged using a bi‐weight robust mean to generate residual chronologies of each sampling group. These steps were performed using the dplR package (Bunn 2010) in R (R Core Team 2015).

We studied Pearson correlations between residual chronologies and different SPEI parameterizations. For a majority of the sampling groups in the period of 1932–2011 (1932–1999 for spruce in Scatlè, 1932–2009 for oak in the corresponding three sites), the most suitable time scale for the SPEI was calculated on a 6‐month time scale for July, that is, integrating the target season from February to July. Calculations of the correlation coefficients between residual chronologies and SPEI for other periods of the year (e.g., time scale of 6 months for September, i.e., integrating April to September) as well as other lengths of the time scales (e.g., time scale of 3 months for August, i.e., integrating June to August) resulted in comparable values due to the overlap of several months and high correlation of consecutive months, respectively, that are considered in different SPEI parameterizations.

By averaging the RWIs of each single tree to a site chronology, information about the variability among the trees is lost. For this reason, we additionally fitted linear mixed‐effects models (LMMs; Pinheiro and Bates 2000) for each sampling group (eq. (1)):

(1) $${\text{RWI}}_{t}=\beta_{0}+\beta_{1}\times {\text{SPEI}}_{t}+b_{0}+b_{1}\times {\text{SPEI}}_{t}+\varepsilon_{t},$$

where RWI_*t*_ is the RWI in the year *t*,* β*_0_ and *β*_1_ are the coefficients of the fixed effects, SPEI_*t*_ is the SPEI in year *t*,* b*_0_ and *b*_1_ are the random effects with $b_{0}∼N\left(0,\sigma_{b_{0}}^{2}\right)$ and $b_{1}∼N\left(0,\sigma_{b_{1}}^{2}\right)$, and *ε*_*t*_ is the residual error in the year *t* with *ɛ*_*t*_ ~ *N*(0, *σ* ^2^). To consider temporal autocorrelation of the RWIs, an autoregressive (AR1) parameter *φ* was added to the model (Pinheiro and Bates 2000): *ɛ*_*t*_ = *φ* × *ɛ*_*t* − 1_ + *η*_*t*_, where *ε*_*t* − 1_ are the residual errors in the previous year (*t* − 1), and *η*_*t*_ is a noise term with zero mean. The nlme package (Pinheiro and Bates 2000) in R (R Core Team 2015) was used to calculate LMMs.

**Figure 1:**
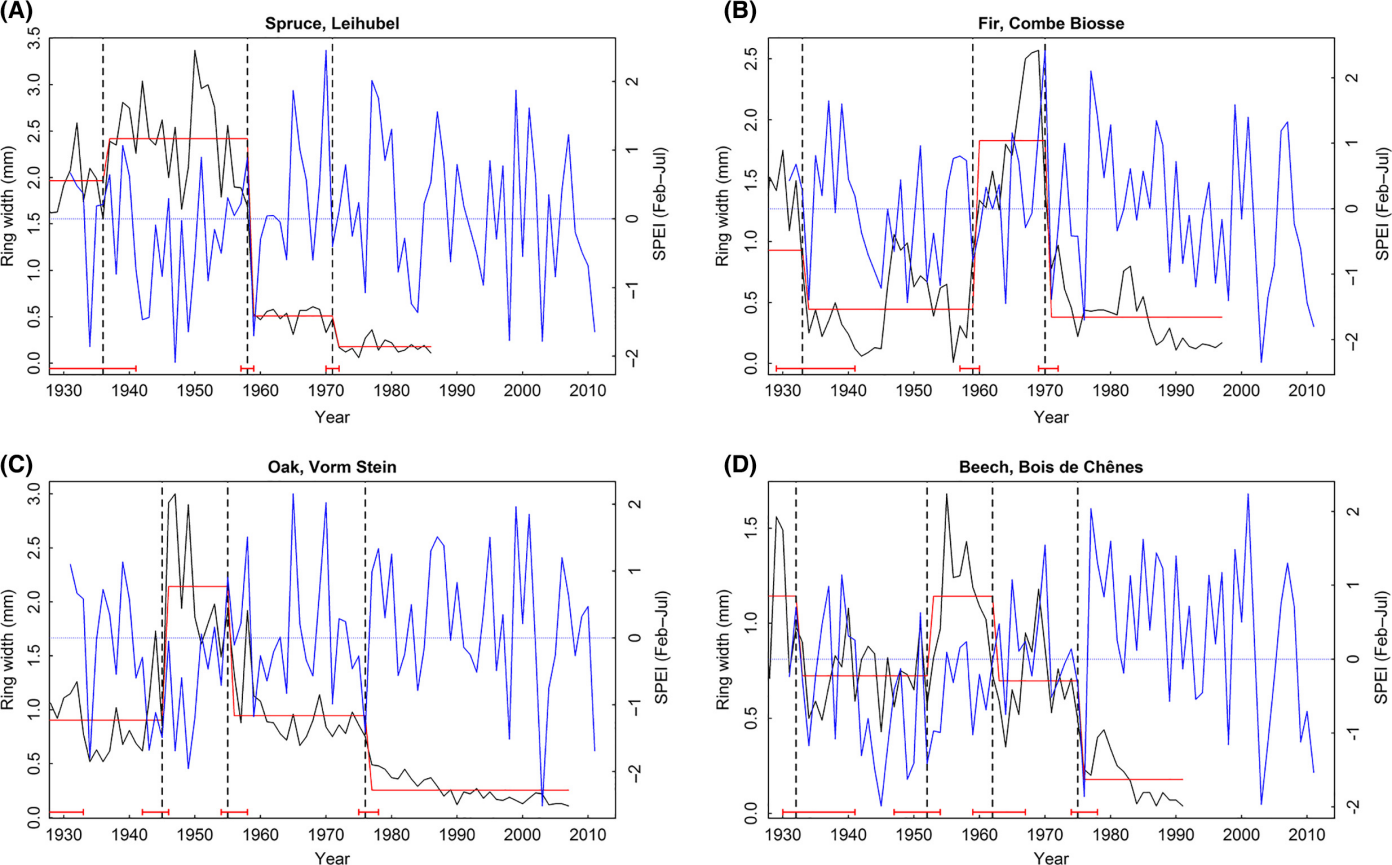
Illustration of breakpoint detection for examples of the four species: (A) spruce, site Leihubel, tree 23, right core, (B) fir, site Combe Biosse, tree 48, left core, (C) oak, site Vorm Stein, tree 50, left core, (D) beech, site Bois de Chênes, tree 09, right core. Shown are annual ring widths (black curves), Standardized Precipitation Evapotranspiration Index (blue curves), breakpoints (vertical dashed black lines), fitted regression lines of linear models (red lines), and confidence intervals of breakpoints (horizontal red lines on bottom).

We did not calculate Pearson correlations or the LMMs to analyze the short‐term growth responses of the dead trees, since dead trees show typically a period of suppressed growth toward the end of the series (e.g., Fig. 1C). During the last few years of a tree's life span, it is possible that rings are missing, which may induce a systematic shift in the tree‐ring pattern. This is unlikely to affect the findings of the long‐term growth responses, since the last abrupt growth changes occurred typically more than 10 or 20 years prior to tree death (Fig. 1).

### Detection of abrupt growth decreases

For each dead tree, we determined when abrupt negative changes in ring widths occurred (Fig. 1). The approach to detect structural changes (Bai 1997; Zeileis et al. 2003) between different growth stages within each series of tree‐ring widths consisted of fitting linear horizontal regression lines over segments of comparable growth rates, which were separated by breaks (i.e., breakpoints). Unlike other well‐established methods in dendrochronology, this model‐based detection method did not require including any predetermined thresholds in growth level changes (cf. Discussion).

For some of the trees, only one core could be crossdated, that is, abrupt growth changes were determined for this core only. For those trees with two crossdated increment cores, the longer series was used for the analysis. Prior comparison of the breakpoints from two corresponding cores of the same tree had indicated in many cases agreement between the detected breakpoints. The analysis was performed using the strucchange package (Zeileis et al. 2002) in R (R Core Team 2015) by applying the function breakpoints (Zeileis et al. 2003), which allows for the simultaneous estimation of multiple breakpoints. As a precondition, the minimum length of the segments between the breakpoints was set to 10 years (e.g., Fig. 1D between 1952 and 1962). Thus, only breakpoints of longer term growth decreases and increases were obtained. Breakpoint calculation with different preconditions such as 15 years or no minimum distance between the breakpoints yielded similar results (cf. Results). The optimum number of breakpoints per series was calculated using the Bayesian Information Criterion (BIC=−2×log(likelihood)+k×log(n), where *k* is the number of parameters and *n* the sample size) by minimizing the residual sum of squares ($\text{RSS}=\sum_{i=1}^{n}{\left(y_{i}-{\overset{¯}{y}}_{i}\right)}^{2}$, where *y*_*i*_ is the *i*‐th observation, and ${\overset{¯}{y}}_{i}$ is the expected value of the *i*‐th observation). Since the function breakpoints assign the breakdates (years when breakpoints occurred) by default to the last year of the previous period (i.e., 1 year before a change to a lower or higher growth level), we shifted the breakdates by +1 year to the first year of the following period (e.g., in Fig. 1A, the negative breakpoint from 1958 was shifted to 1959). This shift is required to test the hypothesis that growth decreases occur in the same year as a drought or are lagged by several years.

Negative breakpoints (i.e., abrupt growth decreases) were determined for the complete time span of every single series. However, since the SPEI was available for the time span between 1932 and 2011 only and due to the investigated lag effect of drought impact from 0 to 5 years, only breakpoints between 1937 and 2011 were considered for further analysis. In a given year (*t*), the number of trees corresponds to *A*_*t*_ and the number of trees in which a negative breakpoint was detected is denoted as *B*_*t*_. The ratio of negative breakpoints (*R*_bp,*t*_) was calculated for each sampling group as follows (eq. (2)):

(2) $$R_{\mathrm{bp},t}=\frac{B_{t}}{A_{t}}\times 100\%.$$

### Distributed lag nonlinear models

The effects of drought (represented by the SPEI) on the occurrence of negative breakpoints were quantified using distributed lag nonlinear models (DLNM, Gasparrini 2011). To our knowledge, DLNMs were only used by Sorg et al. (2015) in the field of environmental sciences. Although site‐ and species‐specific time scales could have been selected for the parameterization of the SPEI, for reasons of comparability we decided to select for all sampling groups the same time scale and season (6 months for July, integrating February–July) as for the short‐term growth response analyses.

To quantify temporally lagged effects of drought on abrupt growth decreases (i.e., occurrence of negative breakpoints), we considered SPEI values over the last 5 years. For each sampling group, we fitted a generalized linear model with a binomial distribution as defined by the probability mass function (eq. (3)):

(3) $$f\left(A_{t};\;B_{t};\;p_{t}\right)=\left(\begin{array}{c} A_{t}\\ B_{t} \end{array}\right)\times p_{t}^{B_{t}}\times {\left(1-p_{t}\right)}^{A_{t}-B_{t}}.$$

The probability *p*_*t*_ of a negative breakpoint to occur in year *t* was modeled with a logit link function (eq. (4)):

(4) $$\ln\left(\frac{p_{t}}{1-p_{t}}\right)=\alpha +\sum_{l=0}^{5}\text{ns}\left({\text{SPEI}}_{t-l};\;2\text{df}\right)+\text{ns}\left(\text{year};\;2\text{df}\right),$$

where *α* is the intercept. A natural cubic spline function (ns) with 2 degrees of freedom (df) allowed for nonlinear relationships and was applied to the SPEI (SPEI_*t* − l_) lagged from 0 to 5 years (*l*). To consider long‐term trends in the number of observations (e.g., fewer observations in more recent times), we also included the variable “year” with a natural cubic spline function and 2 df. In a further step, odds ratios of observing a negative breakpoint were predicted for all sampling groups for the complete range of the SPEI values of each site and lags from 0 to 5 years. Odds ratios are the ratio of the odds (*p*_*t*_/(1 − *p*_*t*_)) of a negative breakpoint to occur at a specific SPEI value to the odds of a negative breakpoint to occur at a reference SPEI, which here refers to the maximum SPEI (i.e., moist conditions) of the time series of each site. The model design was thus based on the assumption that no negative breakpoints are expected under the moistest conditions. Calculations were performed using the packages dlnm (Gasparrini 2011) and splines in R (R Core Team 2015).

We did not analyze the long‐term growth responses of the living trees because (1) only dominant living trees were sampled, that is, the resulting signal would not represent the whole range of social status unlike the dead trees; (2) the sample sizes of the living trees are smaller than those of the dead trees (Table 2) resulting in a lower number of breakpoints; and (3) the tree‐ring series of the living trees do not include the complete life span unlike the dead trees (i.e., both phases of regular growth and phases of reduced growth before tree death are covered by dead trees).

## Results

### Growth patterns of dead trees and death dates

Ring widths of the dead trees varied considerably within the twelve sampling groups (Table 2) due to variability in DBH (5.0–92.0 cm) and differences in canopy positions (suppressed to dominant trees were sampled) as well as among the sampling groups due to different site and climatic conditions (Table 1). Many trees showed decreasing growth trends or periods of reduced growth rates over several years or decades prior to death (cf. Fig. 1). Site‐specific average tree growth rates ranged between 0.76 and 1.19 mm/year and featured high within‐site variability (Table 2). Tree life spans (i.e., cambial age at a height of 1 m) varied from 37 years (fir in Leihubel) to 415 years (spruce in Scatlè), with an average per sampling group between 60.9 and 168.3 years (Table 2). Estimated year‐of‐death dates based on the outermost ring ranged between 1925 and 2013, which explains the shrinking sample size with time within each sampling group.

### Short‐term growth response to climate

**Figure 2:**
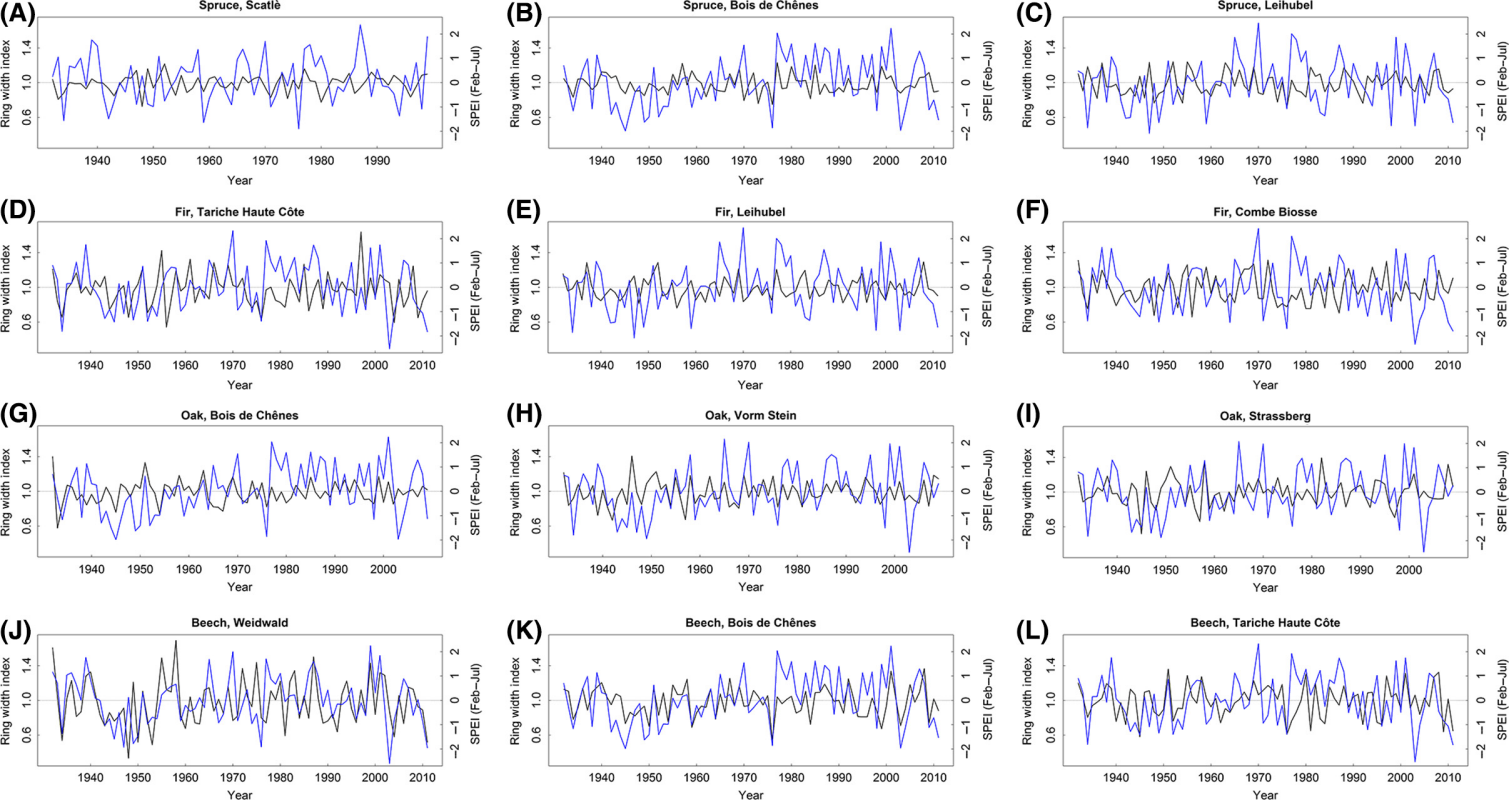
Short‐term growth response to drought. Time series plots of ring‐width index (residual chronology, black curve) and Standardized Precipitation Evapotranspiration Index (6‐months time scale from February–July, blue curve) for the periods 1932–2011 (1932–1999 for spruce in Scatlè), and 1932–2009 for oak in Bois de Chênes, Vorm Stein and Strassberg. Panels within species are listed in ascending order of forest reserve codes (cf. Table 1). The correlation coefficients are shown in Table 3.

Pearson correlation coefficients between residual chronologies and the SPEI from July (integrating February–July) ranged between 0.2 and 0.5 (Fig. 2, Table 3) for the five driest sites (Weidwald, Bois de Chênes, Vorm Stein, Tariche Haute Côte, Strassberg; Table 1). Except for oak in Bois de Chênes, the correlations for these sites were significantly positive, that is, beech in Weidwald, Bois de Chênes and Tariche Haute Côte; oak in Vorm Stein and Strassberg; spruce in Bois de Chênes; and fir in Tariche Haute Côte (Table 3). At the three moistest sites (Combe Biosse, Scatlè and Leihubel; Table 1), which included spruce and fir only, the correlations were not significant.

**Table 3:** Pearson correlations between residual chronologies and Standardized Precipitation Evapotranspiration Index (SPEI) and description of linear mixed‐effects models (LMMs; eq. (1)) between ring‐width indice and SPEI of every sampling group

Sampling group^1^	Pearson correlation (estimate ± SE [*P*‐value])	LMM		
		Fixed effects *β*_1_ (estimate ± SE [*P*‐value])	Random effects *b*_1_ (SD *σ*_*b*1_)	Autoregressive parameter *φ*
Spruce 12	0.036 ± 0.12 (0.77)	0.010 ± 0.0063 (0.10)	0.0030	0.354
Spruce 14	0.39 ± 0.10 (<0.001)***	0.063 ± 0.0098 (<0.001)***	0.027	0.374
Spruce 24	0.048 ± 0.11 (0.67)	−0.0035 ± 0.0070 (0.61)	0.00081	0.382
Fir 22	0.24 ± 0.11 (0.03)*	0.060 ± 0.010 (<0.001)***	0.00089	0.510
Fir 24	−0.040 ± 0.11 (0.72)	−0.0040 ± 0.0078 (0.64)	0.012	0.473
Fir 39	0.029 ± 0.11 (0.80)	0.013 ± 0.0068 (0.060)	0.00017	0.525
Oak 14	0.20 ± 0.11 (0.085)	0.035 ± 0.0083 (<0.001)***	0.032	0.332
Oak 20	0.23 ± 0.11 (0.040)*	0.036 ± 0.0052 (<0.001)***	0.00033	0.302
Oak 30	0.23 ± 0.11 (0.046)*	0.045 ± 0.0065 (<0.001)***	0.0010	0.419
Beech 11	0.44 ± 0.10 (<0.001)***	0.12 ± 0.013 (<0.001)***	0.0052	0.337
Beech 14	0.50 ± 0.098 (<0.001)***	0.061 ± 0.010 (<0.001)***	5.5e‐6	0.337
Beech 22	0.32 ± 0.11 (0.0040)**	0.071 ± 0.0089 (<0.001)***	0.00084	0.306

SE, standard error; SD, standard deviation.

^1^Species and forest reserve code (cf. Table 1).

Significance levels: *P < 0.05; **P < 0.01; and ***P < 0.001.

The outcomes of the LMMs (eq. (1)) confirmed the results from the correlation analysis above. Significant positive effects of the SPEI on individual RWIs were found for the same sites that showed significant positive Pearson correlations; in addition, a significant effect was found for oak in Bois de Chênes (Table 3). At some sites, the between‐tree responses varied relatively strongly within the sampling groups as revealed by the relatively large random effects b_1_ (e.g., for spruce and oak in Bois de Chênes; Table 3).

### Relationship between drought and abrupt growth decreases

**Figure 3:**
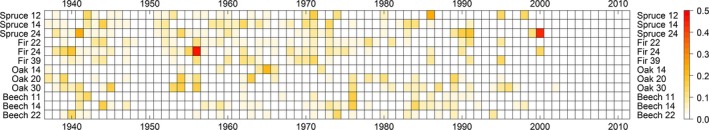
Ratio of negative breakpoints (eq. (2)) per sampling group (cf. Table 2) from 1937 to 2011. Species are listed in ascending order of sampling groups.

The occurrence of negative structural changes in tree growth levels varied temporally across the 12 sampling groups (Fig. 3). From 1937 to 2011, 0–46% of the trees per sampling group and year experienced a negative breakpoint (see eq. (2)). On average over the complete range, 2.6% negative breakpoints per year were detected. Results of breakpoint calculations with different preconditions such as 15 years or no minimum distance between the breakpoints yielded comparable results (cf. Supporting information, Figs. S2, S3).

**Figure 4:**
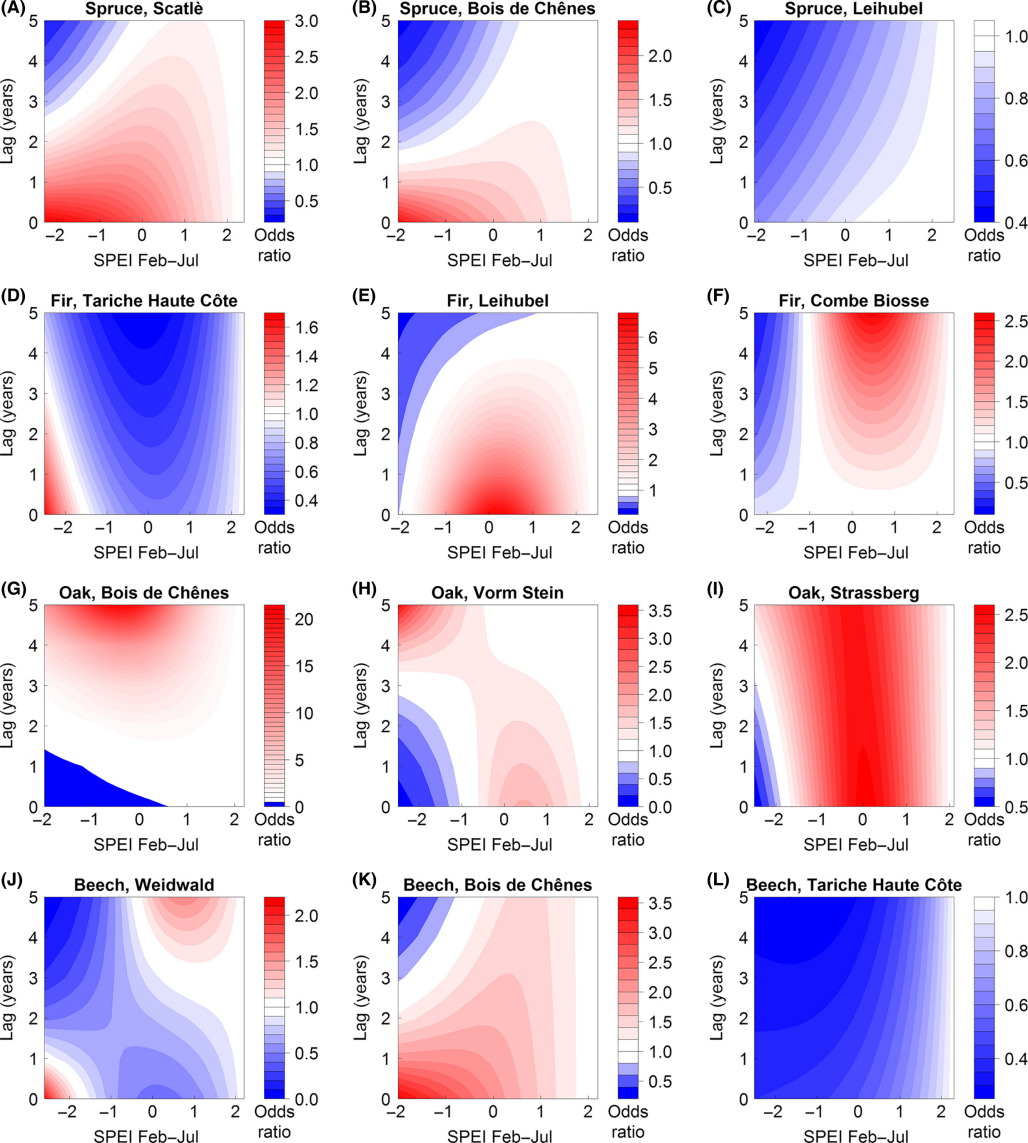
Predicted odds ratios of a negative breakpoint to occur at different Standardized Precipitation Evapotranspiration Index (SPEI) values (range of *x*‐axis based on site‐specific SPEI values) with lags from 0 to 5 years (*y*‐axis). Odds ratios equal 1 at maximum SPEI on each site (reference value). Panels within species are listed in ascending order of forest reserve codes (cf. Table 1).

The DLNMs (see eqs. (3) and (4)) between the SPEI and the negative breakpoints yielded site‐ and species‐specific results. Predictions of the odds ratios (i.e., odds of a negative breakpoint to occur at a specific SPEI value to the odds of a negative breakpoint to occur at the maximum SPEI value) for the sampling groups showed three major patterns (Fig. 4).

**Figure 5:**
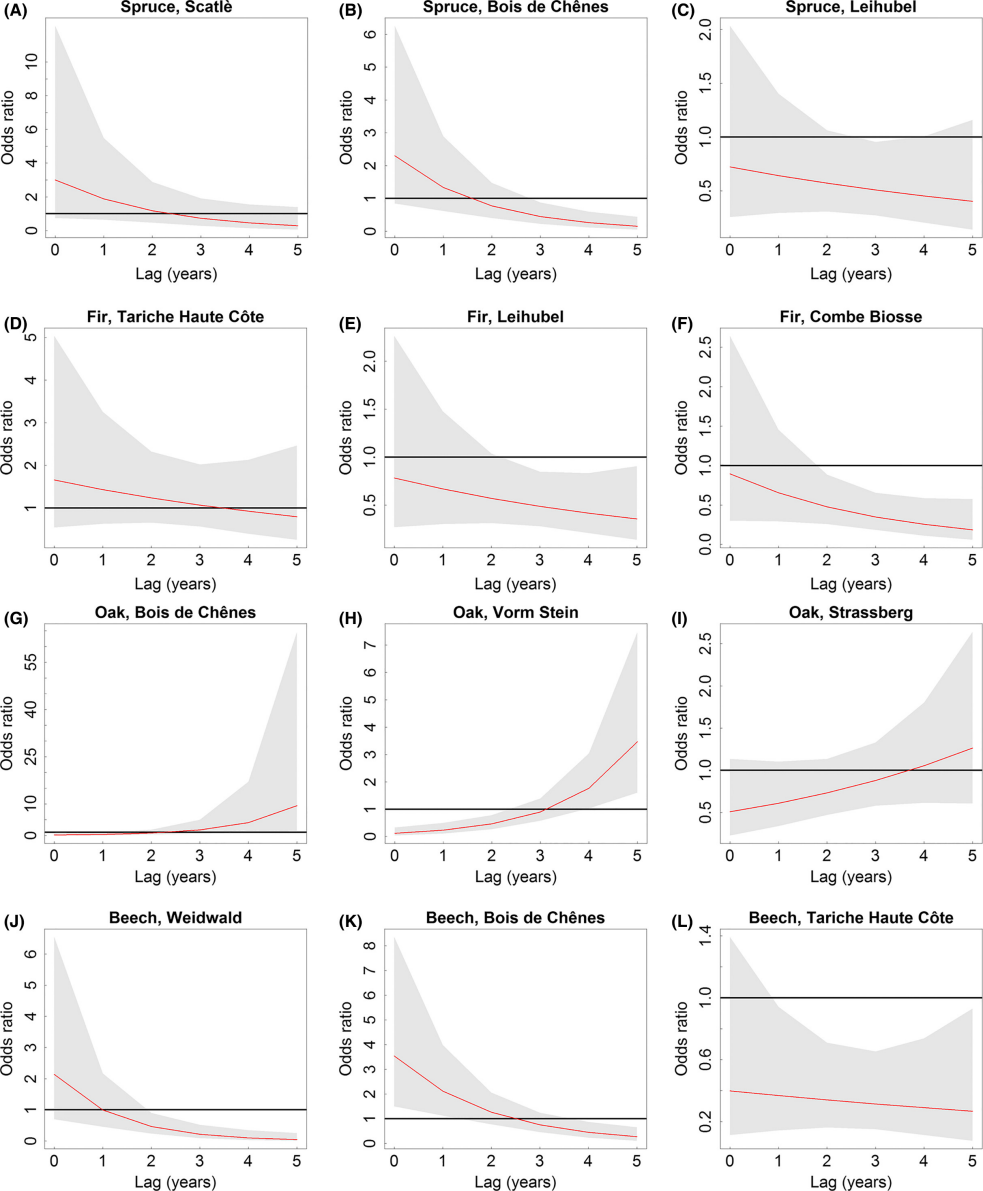
Predicted odds ratios (red curve) including 95% confidence intervals (gray) for site‐specific minimum Standardized Precipitation Evapotranspiration Index values (i.e., most severe droughts) from lag 0 (year of drought event) to lag 5 (5 years after drought event). Panels within species are listed in ascending order of forest reserve codes (cf. Table 1).

First, a trend of higher odds ratios (up to 3.5) at negative SPEI values (i.e., dry conditions) and lags from 0 to 2 years were found for spruce in Scatlè and Bois de Chênes, fir in Tariche Haute Côte, and beech in Weidwald and Bois de Chênes (Fig. 4A, B, D, J, K). However, the odds ratios predicted for the lowest SPEI (i.e., most severe droughts) were significant only for beech in Bois de Chênes (Fig. 5K). Three to 5 years after the most severe droughts, the odds ratios decreased to values below one, indicating significantly lower odds ratios for spruce in Bois de Chênes and beech in Weidwald and Bois de Chênes (Fig. 5B, J, K). Second, oak in Bois de Chênes, Vorm Stein and Strassberg showed increased odds ratios 3–5 years following the most severe droughts, whereas for lags of 0–2 years the odds ratios were below one (Fig. 4G–I). In Bois de Chênes and Vorm Stein, odds ratios were significantly below one during the year of drought and shortly thereafter, whereas they significantly increased after a lag of four to 5 years (Fig. 5G–I). Third, the remaining sampling groups showed less clear patterns, i.e., either decreased odds ratios (for beech in Tariche Haute Côte and spruce in Leihubel) and increased odds ratios at medium and moist conditions, respectively (for fir in Leihubel and in Combe Biosse; Fig. 4C, E, F, L). The predictions for these four sampling groups under the most severe droughts at least partly showed significantly decreased odds ratios after lags of 1, 2 or 3–5 years (Fig. 5C, E, F, L).

Calculations of additional DLNMs with different definitions of the predictor (e.g., SPEI from August (integrating June to August) or response variables (e.g., breakpoints calculated without any preconditions such as minimum distance) resulted in similar distributions of the odds ratio for a majority of the sites (cf. Figs. S4, S5).

## Discussion

We investigated short‐ to long‐term growth responses to drought at the population level and at the level of individual trees (cf. Carrer 2011). The range of these responses is wide, even within the same species in one stand, reflecting the strong spatial variability of environmental conditions in structurally diverse forest stands. Importantly, we complement the widely used analysis of correlations between climate variables (e.g., drought) and tree‐ring chronologies with (1) regression models of short‐term drought effects on RWIs of individual trees and (2) a probabilistic analysis of drought effects on longer term abrupt growth decreases, which accounts for both lagged effects and nonlinear relationships between drought and abrupt growth decreases.

### Short‐term growth response to climate

Analysis of the correlations between residual chronologies and the SPEI (July) yielded significantly positive relationships for most sites, and only a few nonsignificant correlations. Likewise for the LMMs, effects of the SPEI on individual RWIs were significantly positive for the same sites. Unlike the analysis of correlations, the LMMs provided evidence that growth responses to drought may differ substantially among trees. Our findings confirm results from Scharnweber et al. (2011), where a strong dependency on water availability in June and July was identified for oak and beech. Significance for oak in Bois de Chênes was detected in the LMMs only, and no significant positive correlation could be found for spruce in Scatlè, fir in Combe Biosse, and both spruce and fir in Leihubel. The sites with significantly positive correlations tend to be drier (annual precipitation < 1230 mm) and warmer (average temperature > 7.7°C) than the other sites (annual precipitation > 1360 mm, average temperature < 6°C; Table 1). Drought periods have a minor influence only on annual growth variability at the moister and cooler sites, which are located above 1200 m. At these elevations, growing periods are shorter and begin later in spring, leading to a lack of significant correlations between annual ring‐width index and the drought index, which was calculated from late winter to early summer (February–July, *P*‐values > 0.55). The analysis of other periods of the year and other lengths of the time scales of the drought indices (e.g., June to August) resulted in nonsignificant (*P*‐values > 0.07) correlation coefficients for two sites above 1200 m (Combe Biosse and Leihubel), whereas the coefficient for Scatlè was significantly negative (*P*‐value = 0.04).

### Detection of abrupt growth changes

Dendrochronological studies that assess the effects of disturbance events on growth patterns have become increasingly popular (Altman et al. 2014). Many of them have focused on abrupt growth increases (“releases”) induced by the mortality of neighboring trees (Rubino and McCarthy 2004; Altman et al. 2014). Common methods to quantify abrupt growth increases include the calculation of percent growth changes over periods that are fixed from 5 to 15 years, which are compared to either previously defined constant thresholds (Nowacki and Abrams 1997) or flexible thresholds that account for prior growth (Black and Abrams 2003). Similarly, analogous methods are used to detect abrupt growth decreases (Das et al. 2007; Gea‐Izquierdo et al. 2014). The model‐based method to detect abrupt growth changes that is used in this study is more flexible, as it allows for variable lengths of periods between abrupt growth changes and does not require the definition of thresholds. This method has been used only a few times in tree‐ring based studies (e.g., Eilmann and Rigling 2012 or Bishop et al. 2015).

We found negative breakpoints to be widely distributed along time, with annual proportions of abrupt growth changes typically ranging between 10 and 25% (Fig. 3), that is, only relatively few trees per species and site showed synchronous growth decreases. The relatively even temporal distribution of breakpoints may be partly affected by the definition of the breakpoints, which are separated by a minimum of 10 years. This restriction results in a conservative detection of structural changes in the individual tree‐ring series, for example, given two consecutive climatic extreme events that both caused sustained growth reductions within a few years, the second growth decrease will pass unnoticed. This same constraint explains the decrease in the number of abrupt growth decreases that were detected after the year 2000 (Fig. 3). In addition, the accuracy of the breakpoint estimations is reduced by the high year‐to‐year variability of growth rates, resulting in wide confidence intervals of the breakdates ranging from 2 to >10 years (cf. Fig. 1).

The maximum breakpoint ratio of 46% occurred in 1956, when 17 of 37 fir trees in Leihubel experienced an abrupt growth decrease. Yet, for spruce at the same site, only one of 25 spruce trees (4%) experienced a negative breakpoint in that year. The SPEI of the corresponding year was 0.05, which represents average conditions. In 1956, February mean temperature was extremely low (−10.9°C) compared to the long‐term mean (−1.6°C, period 1932–2011), including one period of six consecutive days with daily mean temperatures below −15°C (February 9–14 in 1956). This indicates that the high vulnerability of fir to frost (Lebourgeois et al. 2010) may have induced this growth reaction in 1956.

### Relationship between drought and abrupt growth decreases

The reaction of individual trees to disturbances and their sensitivity to climate extremes depend strongly on factors such as their social status, crown properties, or age (Mérian and Lebourgeois 2011). We found a wide variety of reactions since we wished to characterize the entire population at each site, and thus have sampled suppressed to dominant trees. Yet, the general trend of higher odds ratios during or after drought years corroborates our hypothesis of drought‐related growth decreases on the longer term perspective, which holds across different size and age classes. The DLNMs do not only provide growth responses to single severe drought years, but consider the complete range of drought conditions for the period of 1937–2011.

In five sampling groups including fir, spruce and beech, increased odds ratios imply abrupt growth decreases that follow immediately or shortly after drought, that is, when low precipitation and high temperatures coincide from late winter to early summer (Figs. 4, 5). The more drought‐tolerant oak shows a somewhat different pattern, with lower odds ratios up to 3 years after extreme drought. Drought resistance has been assessed to be higher for oak than for the other three species (Pretzsch et al. 2013; Zang et al. 2014), but our results suggest that oak may experience indirect drought effects as lagged growth decreases, since the odds ratios increase four to 5 years following severe drought (Fig. 5G–I). The initial lack of sensitivity to drought events and the lagged response of oak may be explained by various morphological and ecophysiological adaptions, such as a deep rooting system (Scharnweber et al. 2013), its ability to strongly reduce stomatal conductance during drought (Bréda et al. 1993), or the ability to shed twigs (cladoptosis) to reduce transpiration (Rust and Roloff 2004; Tulik 2014). The latter may be particularly important for the lagged response to drought, since leaf area is reduced for several years and resources need to be re‐allocated to the growth of twigs and leaves, such that less carbon is available for stem growth.

In Bois de Chênes, oak coexists with beech and spruce. Our results suggest that – during and shortly after severe drought – beech and spruce are more likely to suffer from drought, and therefore oak growth may profit indirectly. Still, oak responds with abrupt growth decreases 4–5 years following drought. The other two oak sites (Vorm Stein and Strassberg) are located more than 200 km from Bois de Chênes, but are found only 5 km from each other. If drought is a major trigger of negative breakpoints, their temporal occurrence should be similarly distributed at nearby sites. In fact, increased ratios of negative breakpoints coincide for several years, for example, at both sites a ratio of 16% was observed in 1954, and in 1956, 12 and 15%, respectively (Fig. 3). In Vorm Stein, there is substantial evidence of fewer breakpoints during drought but an increase after some years with comparably narrow confidence intervals of the predictions (Fig. 5H). In Strassberg, the same pattern was predicted, but confidence intervals were somewhat wider. We thus suggest that factors other than drought have contributed to growth decreases in Strassberg.

In Tariche Haute Côte, a productive and almost pure *Fagus*‐*Abies* stand, fir showed increased odds ratios of abrupt growth decreases following severe drought, while for beech the odds ratio 0–5 years after drought was permanently below one. However, both odds ratios did not differ significantly from one at the lowest SPEI value (−2.5) in the drought year. For beech, these low odds ratios imply that structural changes in tree growth were unlikely to be induced only by drought, that is, other factors must have induced these growth decreases.

In Leihubel, no short‐term growth response to drought periods from February to July was identified for spruce and fir (correlation coefficients of 0.048 and −0.040; Fig. 2C and E). These findings are reflected in the lack of abrupt growth decreases during and shortly after severe drought years. Contrary to the response of spruce in Leihubel, which is somewhat moister in spring and early summer than Scatlè (Table 1), but similar to the response of spruce in Bois de Chênes, high odds ratios of spruce in Scatlè showed an increased risk during and shortly after drought. Our findings confirm recent results that emphasize increasing sensitivity of growth response to moisture deficit for spruce (Lévesque et al. 2013; Boden et al. 2014).

The exceptional pattern of predicted odds ratios in Combe Biosse indicates that there is no relationship between drought and abrupt growth decreases for fir at this site. The lack of correlation between the residual chronology and SPEI implies that other limiting factors have to be considered for fir in Combe Biosse, possibly frost, as in Leihubel. Similarly to the extremely low February temperatures in Leihubel in 1956, temperatures in Combe Biosse were much lower than the long‐term mean temperature in years with high breakpoint ratios. In 1956 (ratio of negative breakpoints of 11%), February mean temperature was −11.1°C (long‐term February mean temperature of −1.5°C); in 1962 (12%), March temperature was −2.5°C (long‐term March mean temperature of 1.3°C), and in 1971 (14%), March temperature was −3.0°C, the latter including four consecutive days of daily mean temperatures below −10°C from March 4 to 7. Unlike drought periods, which develop over several months (Vicente‐Serrano et al. 2010), the potential effects of frost cannot be readily considered in the DLNMs. Extreme frosts over just a few days may have severe implications for trees (Schweingruber et al. 1990; Lebourgeois et al. 2010). Time scales of frost periods and minimum temperatures causing long‐term damage are more variable and additionally depend on phenological factors (Dittmar et al. 2006); hence selecting frost events as potential causes of abrupt growth decreases is more challenging than selecting drought events.

### Additional environmental influences on growth

Besides drought and frost, there is a wide range of environmental influences that can incite abrupt negative growth changes. We attempted to avoid as many of them as possible by adapting our study design. Recently damaged trunks (e.g., by wind and snow breakage or rockfall scars) were not selected for sampling. Mechanical damages caused by rockfall are very unlikely in the flat terrain of Bois de Chênes, Vorm Stein and Strassberg, but may occur occasionally in the partially steep terrain of Leihubel, Combe Biosse, Weidwald, and Tariche Haute Côte. Both rockfall and avalanches may be common in the steep subalpine forest Scatlè. However, the selected trees did not show apparent damage by rockfall or avalanches. To exclude the potential impact of massive snow cover on radial growth, we visually compared maximum daily and maximum accumulated snowfall data per year from nearby climate stations of MeteoSwiss of the past 50–80 years with the breakpoint ratios of all sampling groups, but did not find any evidence of snow impact. Windstorms have been related to abrupt changes in ring widths (Hadley and Knapp 2011). The dead trees we selected were not visibly affected by windsnapping, however, if former breakage of the treetop was gradually overgrown, windstorms may also have incited structural growth changes in our sampling. Wildfires as another potential trigger of abrupt growth decreases are uncommon in all our study sites and were neither reported in the archives nor were residues in the stands found. The impact of the European spruce bark beetle (*Ips typographus*) on abrupt growth decreases of Norway spruce is negligible, since in our sample we did not observe the typical sharp growth decline immediately before tree death, which is expected following bark beetle attacks. Defoliating insects are unlikely to occur for the selected species in Switzerland, but may be more important for other species.

## Conclusions

The investigation of correlations between tree‐ring chronologies and a drought index complemented with a quantification of the effect of drought on abrupt growth decreases provides species‐ and site‐specific short‐ and long‐term growth responses to drought. Particularly at arid sites, the selected drought index from late winter to early summer correlates significantly positive with interannual growth variability, that is, growth is reduced during dry periods and increased during moist periods. Our analyses further imply that the relationship between severe climate events such as prolonged droughts and abrupt growth decreases may be revealed by combining a model‐based approach to detect structural growth changes with DLNMs. In many cases, the short‐term response to drought is reflected in an increased risk of abrupt growth decreases during and following drought. However, there are between‐ and within‐species differences in the intensity of the growth responses to drought as reflected by the differing odds ratios. Although several other climatic and nonclimatic factors may affect tree decline, trees across different developmental stages are susceptible to severe drought. Abrupt growth decreases of fir, spruce and beech are lagged by up to 2 years, while oak may react only after 4–5 years. Identifying the onset of a period of reduced growth is crucial for an improved understanding of tree mortality processes. Hence, enhancing this statistical framework by considering additional environmental influences will contribute to a more comprehensive understanding of inciting factors that lead to declining vigor, which may eventually lead to tree death.

## Conflict of Interest

None declared.

## Supporting information


**Figure S1.** Histograms of the twelve sampling groups indicating the number of analyzed dead trees per DBH class in classes of 5 cm.

**Figure S2.** Ratios of negative breakpoints per sampling group from 1937 to 2011 without minimum distance between breakpoints in each single series. Species are listed in ascending order of sampling groups.

**Figure S3.** Ratios of negative breakpoints per sampling group from 1937 to 2011 with a minimum distance of 15 years between breakpoints in each single series. Species are listed in ascending order of sampling groups.

**Figure S4.** Predicted odds ratios of a negative breakpoint to occur, calculated with the SPEI on a 3‐month time scale for August (integrating June–August) as predictor variable (cf. Fig. 4 with SPEI for July [integrating February–July]).

**Figure S5.** Predicted odds ratios of a negative breakpoint to occur, calculated with breakpoints without minimum distance (Fig. S2) as response variable (cf. predicted odds ratios in Fig. 4 with minimum distance of 10 years).
