# Supplementary figures and images for: Quantifying the effects of drought on abrupt growth decreases of major tree species in Switzerland
Source: Ecol Evol. 2016 Apr 20;6(11):3555–70. doi: 10.1002/ece3.2146 (PMC5513292; doi:10.1002/ece3.2146)

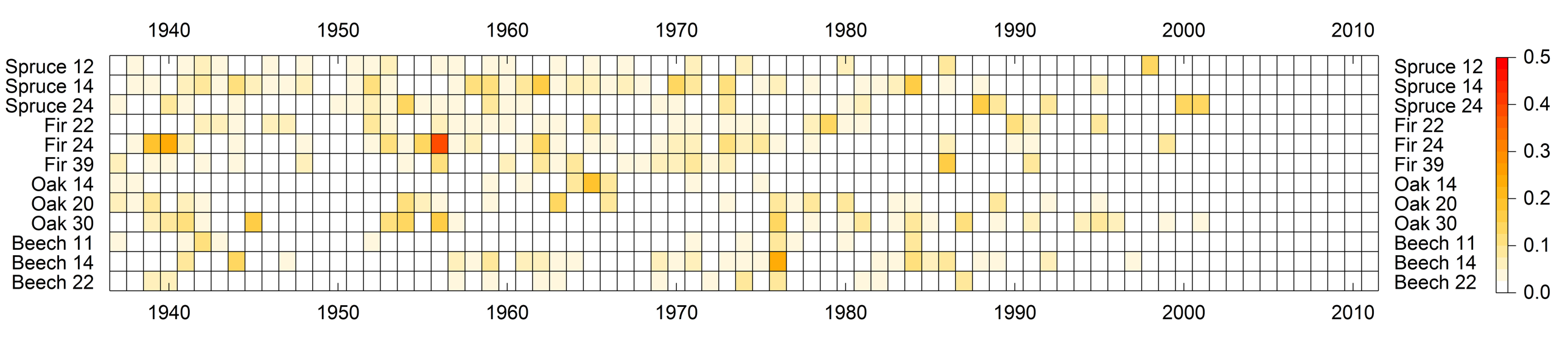

Supplement: Supplementary file 2 — Figure S2. Ratios of negative breakpoints per sampling group from 1937 to 2011 without minimum distance between breakpoints in each single series. Species are listed in ascending order of sampling groups. [file ECE3-6-3555-s002.tif]

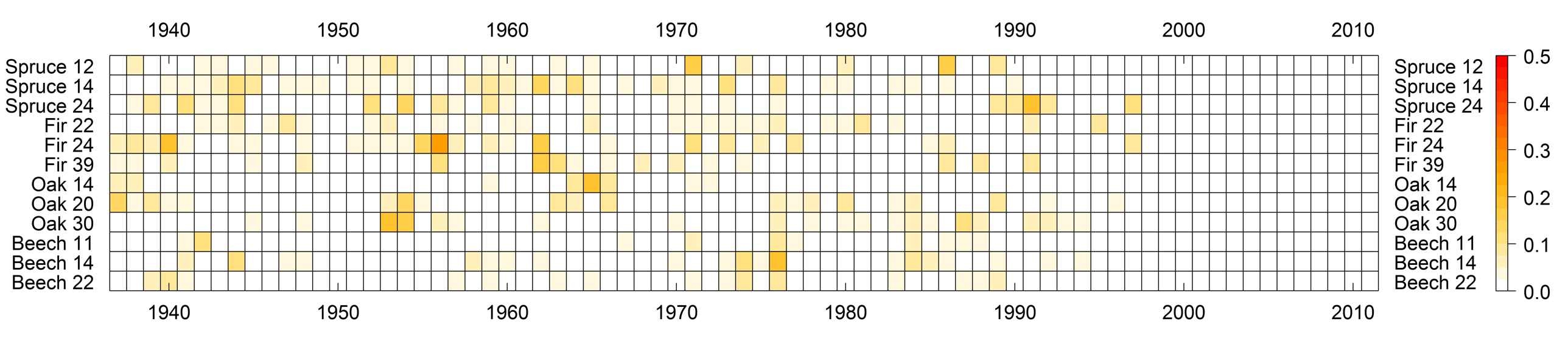

Supplement: Supplementary file 3 — Figure S3. Ratios of negative breakpoints per sampling group from 1937 to 2011 with a minimum distance of 15 years between breakpoints in each single series. Species are listed in ascending order of sampling groups. [file ECE3-6-3555-s003.tif]

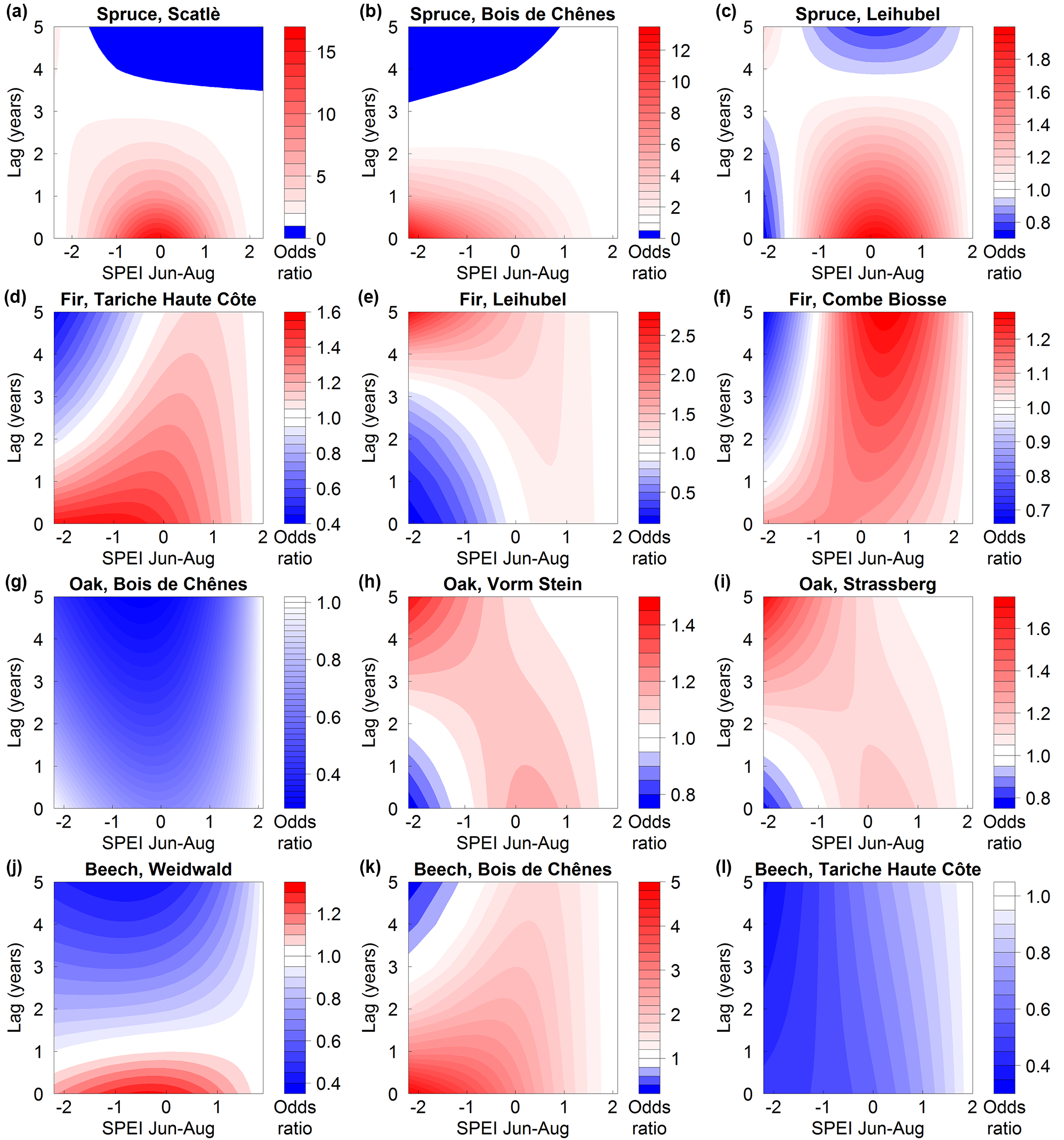

Supplement: Supplementary file 4 — Figure S4. Predicted odds ratios of a negative breakpoint to occur, calculated with the SPEI on a 3‐month time scale for August (integrating June–August) as predictor variable (cf. Fig. 4 with SPEI for July [integrating February–July]). [file ECE3-6-3555-s004.tif]

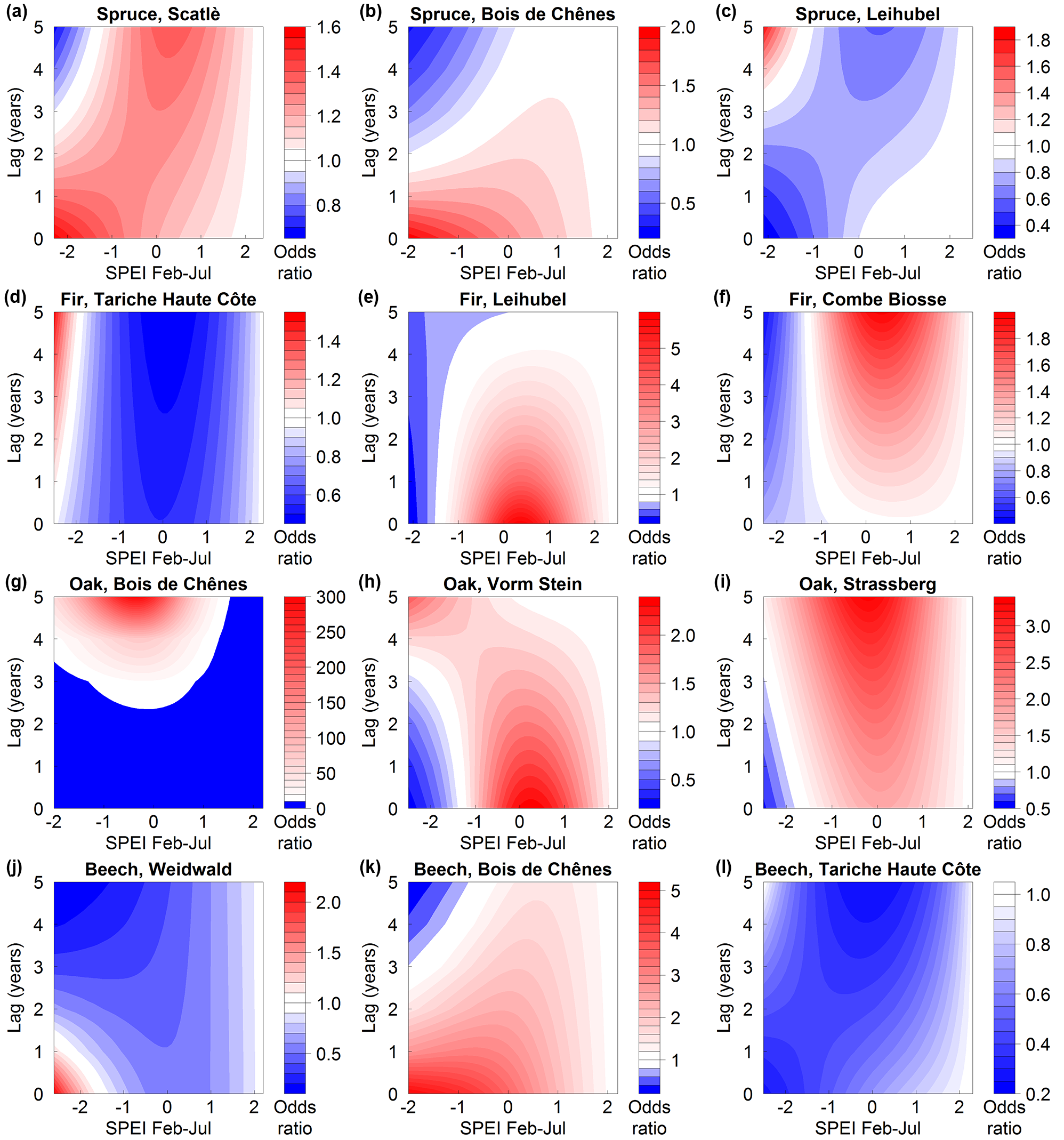

Supplement: Supplementary file 5 — Figure S5. Predicted odds ratios of a negative breakpoint to occur, calculated with breakpoints without minimum distance (Fig. S2) as response variable (cf. predicted odds ratios in Fig. 4 with minimum distance of 10 years). [file ECE3-6-3555-s005.tif]
